# The Abundance and Diversity of Fungi in a Hypersaline Microbial Mat from Guerrero Negro, Baja California, México

**DOI:** 10.3390/jof7030210

**Published:** 2021-03-12

**Authors:** Paula Maza-Márquez, Michael D. Lee, Brad M. Bebout

**Affiliations:** 1 Exobiology Branch, NASA Ames Research Center, Moffett Field, CA 94035, USA; Mike.Lee@nasa.gov (M.D.L.); brad.m.bebout@nasa.gov (B.M.B.); 2 Blue Marble Space Institute of Science, Seattle, WA 98104, USA

**Keywords:** hypersaline microbial mat, fungi, bacteria, quantitative PCR, metagenomics

## Abstract

The abundance and diversity of fungi were evaluated in a hypersaline microbial mat from Guerrero Negro, México, using a combination of quantitative polymerase chain reaction (qPCR) amplification of domain-specific primers, and metagenomic sequencing. Seven different layers were analyzed in the mat (Layers 1–7) at single millimeter resolution (from the surface to 7 mm in depth). The number of copies of the 18S rRNA gene of fungi ranged between 10^6^ and 10^7^ copies per g mat, being two logarithmic units lower than of the 16S rRNA gene of bacteria. The abundance of 18S rRNA genes of fungi varied significantly among the layers with layers 2–5 mm from surface contained the highest numbers of copies. Fifty-six fungal taxa were identified by metagenomic sequencing, classified into three different phyla: *Ascomycota, Basidiomycota* and *Microsporidia*. The prevalent genera of fungi were *Thermothelomyces, Pyricularia, Fusarium, Colletotrichum, Aspergillus, Botrytis, Candida* and *Neurospora*. Genera of fungi identified in the mat were closely related to genera known to have saprotrophic and parasitic lifestyles, as well as genera related to human and plant pathogens and fungi able to perform denitrification. This research suggests that fungi in the mat may participate in nutrient recycling, modification of community composition through parasitic activities, and denitrification.

## 1. Introduction

Microbial mats have been defined as “laminated microbial communities that generally develop in aqueous environments under conditions that exclude fauna” [1]. These communities have been considered to be the first ecosystems on Earth and are thought to have played a fundamental role in the composition of the atmosphere, releasing oxygen, hydrogen and methane [2]. Microbial mats are found in many aquatic environments and are typically detected under extreme conditions, such as hypersaline or hydrothermal environments [3]. Microbial mats are comprised of microorganisms from all three domains of life (Bacteria, Eukarya and Archaea) and viruses [4]. Gradients in light, O_2_, pH, CO_2_ and hydrogen sulfide concentrations create a biological stratification where different taxonomic and functional groups of microorganisms can be distinguished in the layers [5] on a millimeter to centimeter scale. In photosynthetic microbial mats, the upper 1–2-mm-thick layer is generally dominated by oxygenic phototrophs, and the primary production of this layer is responsible for the growth of heterotrophic organisms in this layer and layers below it. Anoxygenic phototrophs may be located below the oxygenic phototrophs and may supply heterotrophs in this layer and layers above and below it with additional fixed carbon which is utilized in both aerobic and anaerobic respiratory pathways. As a result of dramatic environmental changes in the mat, driven by both oxygenic and anoxygenic photosynthesis over the 24 h (diel) cycle, it has been suggested that microorganisms living in microbial mats (individually and as a collective) are necessarily metabolically versatile [6]. Previous studies [7,8] have postulated that the complexity of the microbial mat community is related to the spatial distribution of microorganisms and niche differentiation correlated with profound physicochemical gradients in the mat.

A number of molecular ecological studies have investigated the Bacterial and Archaeal composition of microbial mats, but far fewer (see [9] for a review) have addressed the diversity of Eukarya, let alone fungal diversity. Although fungi were first reported in microbial mats in the 1980s [10], the first diversity study (based on isolation and cultivation techniques) in hypersaline microbial mats occurred in 2006 [11]. Despite the fact that fungi have been documented in many extreme environments, the diversity and roles of these organisms in microbial mats have been largely ignored [9,11]. Factors such as lack of fungal reference genomes [12], biases introduced in the coverage of fungal phyla [13] or limitations related to designing universal DNA primers [14] have contributed to limitations of the knowledge of fungi.

Microbial mats collected from the Guerrero Negro field site are well-studied and are reported to be among the most complex and diverse microbial ecosystems known [7]. The goal of this study was to obtain a more comprehensive description of the fungal diversity and abundance in a hypersaline microbial mat collected near Guerrero Negro, Baja California Sur, México. The diversity and abundance of fungi were evaluated in the mat at 1 mm resolution in seven different layers. A combination of qPCR and metagenomic sequencing was employed to quantify and study the diversity of fungi at various depths in the mat.

## 2. Materials and Methods

### 2.1. Microbial Mat Samples Collection

The microbial mat examined in this study was located in a concentration area in salterns managed by Exportadora de Sal S.A. (ESSA), Guerrero Negro, Baja California Sur, México (ESSA A4; 27°41′15.1” N 113°54′52.1” W). Samples were collected at 9 0900 h on 16th June, 2019 from concentration Area 4 (along the dike between Area 4 and Area 5), as previously described [15,16]. The salinity of the brine in the concentration area at the time of collection of the mats was 125 ppt, the temperature was 24.4 °C, the ammonium concentration was 0.12 µM, the pH 8.3 and the dissolved oxygen concentration was 7 mg/L. Nitrate concentrations were below the limit of detection (<0.5 µM).

A total of 18 mat samples (20 × 25 cm in area and with a thickness of 5 cm) were collected and transported to NASA Ames Research Center (Moffett Field, CA, USA). Three replicate cores from the collected mats were taken using a stainless-steel corer having an inner diameter of ~1 cm as previously described [17]. Mat cores were placed into plastic centrifuge tubes (Falcon^®^, Corning, Corning, NY, USA) capped, and immediately frozen in liquid nitrogen. Vertical layers of frozen mat were sectioned at one-millimeter intervals using sterile scalpels. For qPCR, seven layers from each of three replicate cores were processed independently. For metagenomic analysis, the first four layers (0–1, 1–2, 2–3 and 3–4 mm from the top of the mat) from an additional 3 replicate cores were pooled for library preparation and sequencing, resulting in a single pooled metagenome for each of the 4 layers.

### 2.2. Oxygen Profiles and Microscope Analysis

Oxygen concentration profiles (three replicates) at the upper top 4 mm of the mat were measured at noon time (1200 h) and at midnight (0000 h) using microsensors in the flumes at NASA Ames Research Center. Glass microelectrodes (Unisense, Aarhus, Denmark) were deployed using micromanipulators and a custom-built positioning and data acquisition system. Electrode signals acquired by a Unisense PA-2000 picoammeter were converted to oxygen concentration with a two-point calibration using air-saturated and nitrogen-bubbled brine. Microscope analysis was performed to differentiate the morphological characteristics of the layers.

### 2.3. DNA Extraction and qPCR Analysis

Total nucleic acids were extracted from each microbial mat layer, using a DNeasy Power Biofilm Kit, (Qiagen, Venlo, The Netherlands), according to manufacturer’s instructions. The quality (A260/A280) and quantity (A260) of extracted genomic DNA was determined with using a nanophotometer (Implen GmbH, München, Germany).

Gene copy numbers of bacteria and fungi were quantified in each 1 mm slice by qPCR. The universal primers pairs 341F-534R (5′-CCTACGGGAGGCAGCAG-3′, 5′-ATTACCGCGGCTGCTGG-3′; 341F-534R, respectively) and FungiQuant (5′-GGRAAACTCACCAGGTCCAG-3′, 5′-GSWCTATCCCCAKCACGA-3′; FungiQuant-F, FungiQuant-R, respectively) were selected to amplify the 16S rRNA and 18S rRNA gene of bacteria and fungi [18,19]. The amplification conditions used were reported previously for bacteria [18] and for fungi [19]. Quantifications were performed using an Illumina Eco™ Real-Time PCR System (Illumina Inc., San Diego, CA, USA). Quantitative amplifications were performed in a total volume of 10 µL as previously described [16]. Each qPCR amplification contained 5 µL of KAPA™ SYBR^®^ FAST qPCR master mix, 500 nM of each primer and 1 µL (30–95 ng) of the template DNA. Absolute quantifications were performed by constructing standard curves using series tenfold dilutions of plasmids harboring PCR-amplified inserts of the targeted marker genes. The efficiency of PCR amplification for all targeted marker genes was 90–100%. All samples were measured in triplicate and negative controls were included in all qPCR and RT-qPCR assays. The number of copies of each targeted gene estimated by qPCR were normalized per g of mat and per ng of nucleic acid.

### 2.4. Metagenomic Sequencing

Library preparation and sequencing were performed by MR DNA–Molecular Research LP, (Shallowater, TX, USA). In this process, 50 ng genomic DNA was used to prepare the libraries with the Nextera DNA Flex preparation kit (Illumina, San Diego, CA, USA) following the manufacturer’s user guide. The samples underwent simultaneous fragmentation and addition of adapter sequences. Libraries were pooled and diluted (to 0.6 nM), and 2 × 150 paired-end sequencing was performed on the NovaSeq system (Illumina, San Diego, CA, USA). Metagenomic sequence data from the 4 depths are available through NCBI at BioProject PRJNA688760

### 2.5. Metagenomic Processing

Conda v.4.9.0 (2020; www.anaconda.com (accessed on 10 March 2021).) was utilized for program installation and environment management. Sequence quality was scanned with FastQC v0.11.9 [20] and reads were trimmed/filtered with trimmomatic v0.39 [21]. Assembly-based approaches were not successful in recovering Eukarya-derived contigs, so we utilized a read-based approach to interrogate as much of the fungal community as possible. Read-based taxonomic classification was performed with Kraken2 [22] v2.0.8_beta, utilizing their standard reference database as built in January 2020, followed by Bracken [23] v2.5 for relative abundance estimation at the species level with default settings other than -r 150 for read-length size. Around 15% of reads were successfully classified, of which ~15,000–28,000 reads (~1%) were classified as fungal; these were utilized herein. This relatively low amount of recovered fungal reads did preclude doing any extensive functional analyses with the metagenomic data. All taxonomic information presented here was derived from this metagenomics data.

The Shannon–Wiener index (*H*’) was calculated using PAST v.3.06 [24]. Heatmaps representing the relative abundance of taxa summarized at the taxonomical ranks of phylum, family and genus were performed using the gplots and RColor-Brewer packages for R v.3.2.0 [25].

### 2.6. Statistical Analysis

IBM SPSS Statistics v.19 (SPSS INC., Chicago, IL, USA; IBM, Armonk, NY, USA) was used to test for the normality of qPCR data using the Shapiro–Wilk’s test. The Kruskal–Wallis and Conover–Iman tests were selected for comparisons among groups of samples across depths, in search of significant differences. A 95% level of significance (0.05) was utilized. Individual taxa contributions to the samples’ overall (dis)similarities were estimated with similarity percentages analysis (SIMPER) [24].

## 3. Results

### 3.1. Microscope Analysis and Oxygen Profiles

**Figure 1:**
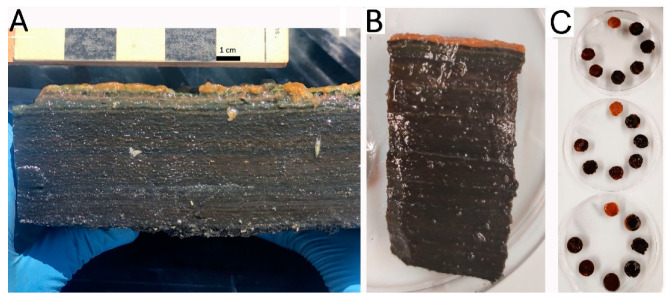
(**A**) Hypersaline microbial mat at the time of collection from a saltern in Guerrero Negro, Baja California Sur, México, scale at top is in centimeters, photo credit: José Q. García Maldonado, (**B**) Higher magnification image of a cross sectional view of a 1 cm diameter core showing the mat laminations, and (**C**) Sections of the core made at 1 mm intervals (3 replicate cores shown in 10 cm Petri dishes).

The mat studied was a 5 cm-thick, dense and laminated structure with a tofu-like texture (Figure 1A). Laminations had markedly different colors (Figure 1B). Sectioning at 1 mm intervals produced sections that did not always correlate with the colors and sizes of the naturally occurring laminations (Figure 1C). However, the 1 mm sections had some unifying physical characteristics. These sections could also be described and differentiated on the basis of their diel ranges in dissolved oxygen, as determined using microelectrodes (Table 1). Layer 1 (0–1 mm from surface; 1 mm thick) was light orange-light-brown in color and composed primarily of diatoms and associated exopolysaccharides. Oxygen concentrations in Layer 1 were somewhat elevated from water column oxygen concentrations during the day, and only slightly lower than water column concentrations at night. This layer was designated “Mostly Oxic” for the purposes of later comparisons. Layers 2 and 3 (1–2 mm and 2–3 mm from surface, respectively) are darkly pigmented and compact, exhibited high oxygen concentrations during the day and low oxygen concentrations at night. Layer 2 had the highest measured oxygen concentrations in any layer. These two layers had the greatest diel changes in oxygen tension changes of any layers examined, and were designated “Oxic and Anoxic I”, and “Oxic and Anoxic II”, respectively. Layer 4 (3–4 mm) contained very low concentrations of oxygen at noon under cloud-free conditions, but is anoxic through much of the diel cycle, particularly on cloudy days, and so was designated “Mostly Anoxic”. Layers 5–7 are completely anoxic.

### 3.2. Abundance of Fungal and Bacterial Derived Sequenced as Determined by qPCR

**Figure 2:**
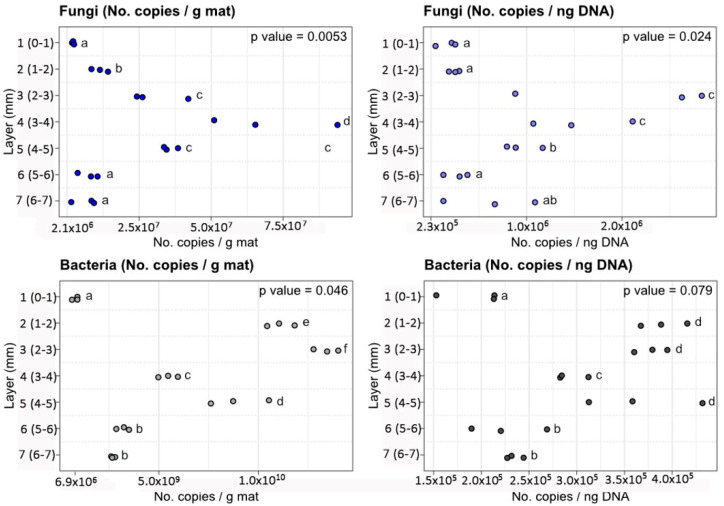
Copies of 18S rRNA fungal (top panels) and 16S rRNA bacterial (bottom panels) genes recovered, per g of microbial mat (left panels) or ng of DNA (right panels), quantified by qPCR in hypersaline microbial mat sections from different depths. Different letters between layers denote statistical differences, according to Conover-Iman and Kruskal–Wallis tests.

Figure 2 shows the numbers of copies of fungi and bacteria recovered in the different layers. For both groups of microorganisms, the numbers of copies were normalized by both g of mat and ng of DNA. Significant differences in the numbers of copies of genes from both target groups were detected between different layers (Kruskal–Wallis and Conover–Iman tests). Similar dynamics in the copy numbers were detected for data normalized both by copy number and by grams of DNA. The ranges of copy numbers are detailed in Table 2.

Fungi were detected in all layers analyzed. Copy numbers of fungi 18SrRNA genes varied in the range 10^6^–10^7^ per g of mat (or 10^5^–10^6^ copies per nanogram of DNA) (Figure 2). The highest number of copies were detected in Layers 3, 4, and 5 (2–5 mm from surface), while layers 1, 2, 6 and 7 (0–2 and 5–7 mm from surface) were characterized by the lowest numbers of copies.

The absolute abundance of bacteria-derived gene copies were in the range of 10^8^–10^10^ per g of mat (10^5^ per ng nucleic acid). Layers 1, 6, and 7 (0–1 and 5–7 mm from surface) contained the lowest number of recovered copies, while Layers 2–5 (1–5 mm from surface) contained the highest number of copies.

### 3.3. Metagenomics

**Figure 3:**
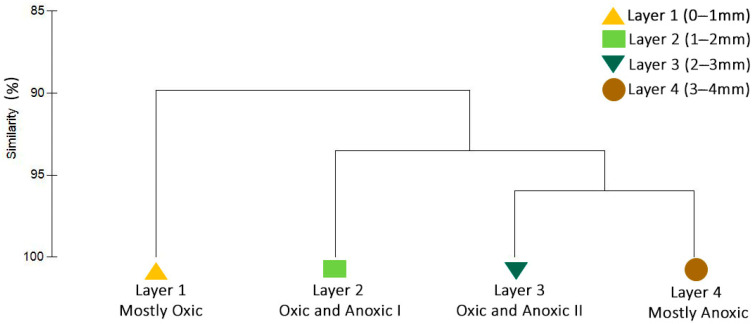
Prevailing oxygen tensions and cluster analysis illustrating the similarity of the fungal communities in samples retrieved from the 4 upper layers. Clustering was generated with the UPGMA algorithm, using Bray Curtis similarities generated from the species-level relative abundance of the metagenomic reads classified as fungi from samples [layer 1 (0–1 mm from surface), layer 2 (1–2 mm from surface), layer 3 (2–3 mm from surface), layer 4 (3–4 mm from surface)].

Supplementary Table S1 presents all read counts and taxonomic classifications. The Shannon-Wiener index (H’) calculated for the fungal community based on the species rank scored >3 for all the layers (average of 3.73). The dendrogram in Figure 3 illustrates the clustering of samples based on Bray Curtis similarities according to the relative abundance of species-level classification. The fungal community structure based on the relative abundance of species-level taxa detected by metagenomic sequencing showed high stability (>90% overall similarity), and there were no significant differences between layers, as confirmed by ANOSIM (R = −0.095) analysis.

**Figure 4:**
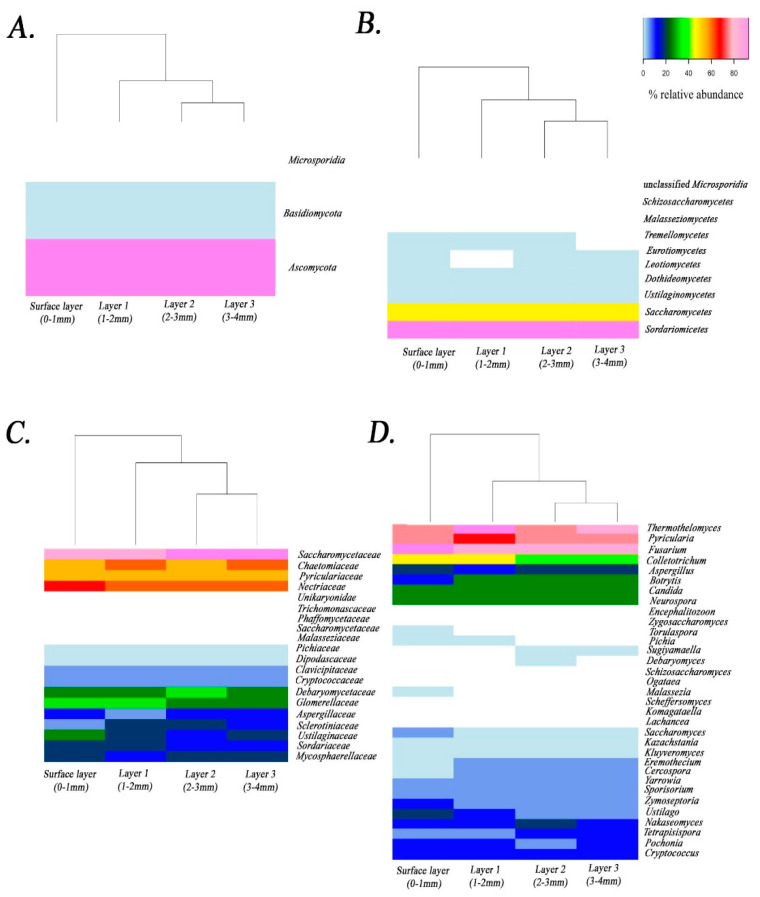
Heatmap showing the relative abundance of fungal taxa at Phylum (**A**), Class (**B**), Family (**C**) and Genus level (**D**) detected by read-based classification of metagenomic sequencing in the different layers [layer 1 (0–1 mm from surface), layer 2 (1–2 mm from surface), layer 3 (2–3 mm from surface), layer 4 (3–4 mm from surface)].

Read-based classifications of fungi identified 3 phyla, 10 classes, 14 orders, 20 families, 33 genera and 56 species. The dominant phylum was *Ascomycota*, followed by *Basidiomycota* and *Microsporidia* (Figure 4A). At the class taxonomic level (Figure 4B), *Sordariomycetes* was the most prevalent, followed by *Saccharomycetes, Leotiomycetes, Dothideomycetes, Ustilaginomycetes, Eurotiomycetes, Tremellomycetes, Schizosaccharomycetes, Malasseziomycetes* and the unclassified *Microsporidia*. The class *Sordariomycetes* comprised six different genera (Table 3). *Saccharomycetes* was the second most abundant class (average of relative abundance > 28%) and the most diverse, with 17 different genera (Table 3). The class *Leotiomycetes* comprised the genus *Botrytis*. *Dothideomycetes* contained two genera: *Cercospora* and *Zymoseptoria*. *Ustilaginomycetes* comprised the genera *Ustilago* and *Sporisorium*. *Eurotiomycetes* contained the genus *Aspergillus*. *Tremellomycetes* comprised the genus *Criptococcus*. *Schizosaccharomycetes* comprised the genus *Schizosaccharomyces*. *Malasseziomycetes* comprised the genus *Malassezia*. Finally, the unclassified *Microsporidia* comprised the genus *Encephalitozoon*.

At the family level (Figure 4C), the dominant families were Saccharomycetaceae, Chaetomiacea, Pyriculariaceae, Nectriaceae and Glomerellaceae. Otherwise, the families Trichomonascaceae, Phaffomycetaceae, Malasseziaceae, Schizosaccharomycetaceae and Unikaryonidae, displayed the lowest relative abundance in the samples.

At the genus level (Figure 4D), the predominant genera were Fusarium, Pyricularia, Thermothelomyces and Colletotrichum, while the genera Debaryomyces, Komagataella, Zygosaccharomyces and Encephalitozoon were less dominant.

The contribution of each species-level taxa to the dis(similarity) between samples was evaluated by SIMPER [24]. The results of this analysis reveal that 6 genera (*Fusarium, Thermothelomyces, Pyricularia, Colletotrichum, Candida* and *Neurospora*) together drive >50% of the global similarity between the different layers (Table 4).

## 4. Discussion

In this study, fungal abundance and diversity were studied at 1 mm resolution using quantitative PCR and metagenomic sequencing. Both methods revealed that fungi are present throughout the top seven millimeters of the mat despite extreme salinity, and large diel fluctuations of oxygen and other physicochemical gradients (light, pH, and nutrients). Fungi are known to be ubiquitous eukaryotic microorganisms and can survive extreme environments of salinity, pH or temperature [11,26,27,28,29].

While fungi have been described from this same microbial mat using molecular techniques [30] and in other microbial mats using isolation and molecular techniques, e.g., in hypersaline mats from Cabo Rojo, Puerto Rico [11] or with morphological and cloning techniques, e.g., in the hypersaline lagoon of Shark Bay, Australia [31], the data reported here are, to our knowledge, the first quantitative abundance data on the vertical distribution of fungi in hypersaline microbial mats. In the discussion that follows, we present some observations about the abundance and diversity of fungi identified in the microbial mat studied here and suggest some possible roles for fungi in these systems.

### 4.1. Abundance of Fungi in the Guerrero Negro Microbial Mat

The abundance of fungi in the Guerrero Negro mat, as assessed by 18SrRNA genes ranged between 10^6^ and 10^7^ copies/g mat. The lowest numbers of fungal and bacterial recovered sequences were detected in Layer 1 (Figure 2, Table 2). Layer 1 is a relatively uncompacted and lightly pigmented layer, composed primarily of diatoms and exopolysaccharides (Figure 1B). The highest number of copies of fungal-derived 18S rRNA sequences were detected between Layers 2–5 (Figure 2, Table 2) (1–5 mm from surface of mat). Due to the extreme variations in oxygen tension across the diel cycle, Layers 2–44 represent an unusual niche for fungi.

While the number of fungi gene copies detected were two to three orders of magnitude lower than those of bacteria, the number of copies were similar to those reported from other complex systems in which fungi have been reported to play an important role. These systems include conventional [32] and nonconventional, (e.g., using a combination of anaerobic/anoxic/aerobic bioreactors in series (A^2^O)) wastewater systems [33]), as well as composting [34] systems.

### 4.2. Diversity of Fungi in the Guerrero Negro Microbial Mat

The Shannon (*H*’) diversity index presented herein based on metagenomic read-based classifications averaged of 3.73. In other microbial ecosystems, based on varying measurements, values of Shannon (*H*’) ranged between 1.45 and 4.5 for conventional and non-conventional wastewater technology systems [33,35], 2 and 4 for a composting system [34], 2.52 and 5.38 for coastal wetland sediment [36], 3 and 5 for desert and mountains [37,38] and 6 and 7 for a soil ecosystem [39]. Though we note that diversity indices applied to sequencing data are heavily influenced at each stage from sample acquisition through to sequence processing, and metrics from different data types and studies are not necessarily comparable.

The relative abundance of fungal taxa recovered with depth (Figure 3) shows that the fungal community is highly stable (>90% similarity) across all layers examined, and across very different physicochemical conditions. *Ascomycota* was the dominant phylum in the community (Figure 4A), in agreement with previous reports of fungi in the same ecosystem [30], in other hypersaline microbial mats [40] and in other diverse natural environments [41]. The sequences of fungi reported from this same microbial mat by Feazel [30] on the basis of a rather more modest sequencing effort than the one presented here, were three clones from a single species, the Ascomycete *Metschnikowia bicuspidate*. It is well documented that *Ascomycota* contains a high number of species with great diversity of lifestyle in many environments [42]. *Microsporidia* was the least abundant phylum of fungi in this microbial mat. *Microsporidia* is diverse group of parasites with highly adapted, sophisticated, and unique infection mechanisms [43]. These fungi have been previously reported in microbial mats from the Artic using metagenomic sequencing [44].

### 4.3. Potential Roles of Fungi in the Guerrero Negro Microbial Mat

The data presented here indicate that the same or very similar fungal taxa, and very similar abundances are present in contiguous layers in the mat. This finding presents the intriguing possibility that the fungi in contiguous layers are, in fact, the same individuals. In this case, the mycelia of individual fungi could connect layers of the mat having distinctly different oxygen (and possibly other) environmental conditions, as has been previously suggested without diversity and abundance data [9]. It was speculated by these same authors that fungi could then perform a role in the equalization of physicochemical gradients in mats. It is certainly the case that fungal hyphae in soils are often reported to be sufficiently long to transit aerobic and anaerobic layers of the microbial mat [45]. An intriguing consequence of this habit is that a hypha of a single individual experiences vastly different physicochemical regimes across its length, presenting a unique set of challenges for the cellular regulation of metabolism of the individual. Whether or not they mediate gradients and/or the exchange of solutes between these layers in mats, as has been well-documented in soils [46] should be the subject of further studies.

Due to their considerable length, fungal hyphae may perform a role in the physical stabilization of cell aggregates between layers in mat as they have been shown to do in other environments. As an example, mycelia in soils can extend from a few microns to several meters long, forming an intricate network [45]. The role of fungi contributing to cell stabilization is also well known in wastewater microbial communities [47].

Perhaps the role most often associated with fungi is the decomposition of organic matter, and it has been suggested that fungi also facilitate nutrient regeneration in microbial mats [9]. The relative abundance and community composition of fungi were substantially similar in the different layers of the microbial mat studied here, suggesting that the remineralization the organic matter and exchange of substrates may occur similarly in all layers examined. In the present study, *Saccharomycetales* was the most abundant order of fungi in the mat (average relative abundance > 28%). It was also the most diverse; phylotypes from 17 different genera were detected (Figure 4D). Members of the order *Saccharomycetales* are known to live as saprobes and at least 1000 species have been described in this single order [48]. The high diversity of *Saccharomycetales* may be evidence the potential of these fungi to contribute to the cycling of diverse substrates in the mat. Taxa closely related to genus *Aspergillus* (order *Eurotiales*) also have been related with nutrient cycling in diverse environments [49] and a recent study [50] described a role for *Aspergillus* in the solubilization of phosphate.

Parasitic fungi are ecologically important in many natural communities and have been shown to regulate the food web, alter the energy flow, and affect community composition [51]. *Microsporidia* and the genus *Encephalitozoon* are unicellular parasites [52] and were detected in this study (Figure 4). *Encephalitozoon* increased the relative abundance in the deep layers (0.26, 0.41, 0.53, 0.61% of relative abundance, respectively). Fungal predation on Cyanobacteria by *Emericellopsis* has been described in microbial mats [53] and in other freshwater ecosystems [51]. We note that as there were no *Emericellopsis* reference genomes in NCBI at the time of this work, these would not have been detected in our samples whether they were present or not.

Fungi well-known to participate in the process of denitrification in other environments [54,55] were also detected in the Guerrero Negro microbial mat. *Aspergillus* and *Fusarium* are among the known fungal denitrifiers which were identified in the Guerrero Negro microbial mat in this study. Denitrifying fungi are known to perform the reduction of nitrate (NO_3_) to nitrite (NO_2_), the reduction of NO_2_ to nitric oxide (NO), and the reduction of NO to nitrous oxide (N_2_O), and the enzymes nitrate reductase, nitrite reductase, and nitric-oxide reductase have been previously reported in several species of fungi [56,57,58]. Somewhat curiously, no fungi have been reported which can catalyze the last step in denitrification, namely the reduction of N_2_O to dinitrogen (N_2_). Interestingly, denitrification in this same microbial mat has been shown, using stable isotopic techniques, to be largely incomplete (stopping at the production of N_2_O rather than N_2_, [59]. N_2_O is among the most potent (298 times more effective than CO_2_) greenhouse gas [60,61]. As previous studies have reported that fungi could perform >18% of denitrification in soils [62] N_2_O emission by fungal denitrification in these environments could be of global importance [63,64]. It is clear that developing efficient mitigation strategies for N_2_O emissions requires the kind of identification of the microbial players provided by studies such as this. Further studies should be performed to enhance our knowledge of fungal contribution of N_2_O by microbial mats.

Finally, we detected a number of genera related to fungal pathogens of plants and humans in the studied microbial mat. *Fusarium*, *Pyricularia*, *Colletotrichum, Botrytis, Ustilago* and *Pochonia* are filamentous fungi which cause several diseases in agricultural crops and natural species [65,66,67,68,69,70]. *Aspergillus, Candida* and *Cryptococcus*, all related to human pathogens were also detected [71].

## 5. Conclusions

The results presented here provide novel findings on the abundance and diversity of fungi in a hypersaline microbial mat. qPCR analysis of the 18S rRNA gene of fungi and the 16S rRNA gene of bacteria revealed that fungi were present throughout the community, with gene copy numbers two logarithmic units lower than bacteria. The greatest number of gene copies attributable to fungi was detected in Layers 3, 4 and 5 (2–5 mm from surface).

On the basis of the data presented here, we postulate that fungi in the Guerrero Negro mat are involved in at least three potential roles: 1) nutrient recycling (based on the prevalence of saprobic taxa), 2) mediating community composition (through taxa exhibiting a parasitic lifestyle), and 3) denitrification (based on the presence of genera related to fungi known to mediate this process). A fourth potential role, that of a physical stabilization of the community, may be inferred, but not conclusively shown, from the similarity of abundance and community composition across millimeter-scale distances, as well as the demonstrated role of fungi in the stabilization of cell aggregation in other natural microbial communities.

## Figures and Tables

**Table 1:** Ranges of oxygen concentration in hypersaline microbial mat from different depths. The depths shown are Layer 1 (0–1 mm from surface), Layer 2 (1–2 mm from surface), Layer 3 (2–3 mm from surface) and Layer 4 (3–4 mm from surface).

Layer, (Depth Below Surface)	Oxygen Concentration (µM)
Layer 1, (0–1 mm)	200–800
Layer 2, (1–2 mm)	0–1200
Layer 3, (2–3 mm)	0–200
Layer 4, (3–4 mm)	Only detectable under very high light
Layers 5–7, (4–7 mm)	not detectable

**Table 2:** Average ± standard deviation of the number of copies (per g of mat and ng of nucleic acid) of the fungi and bacteria in different layers of mat.

	A. Fungi		B. Bacteria	
Layer, (Depth Below Surface)	Copies/g mat	Copies/ng DNA	Copies/g mat	Copies/ng DNA
Layer 1, (0–1 mm)	2.47 × 10^6^ ± 3.42 × 10^5^	1.84 × 10^5^ ± 1.10 × 10^5^	8.27 × 10^8^ ± 1.93 × 10^8^	1.85 × 10^5^ ± 4.31 × 10^4^
Layer 2, (1–2 mm)	1.17 × 10^7^ ± 2.88 × 10^6^	2.57 × 10^5^ ± 5.46 × 10^4^	1.12 × 10^10^ ± 9.80 × 10^8^	3.93 × 10^5^ ± 3.44 × 10^4^
Layer 3, (2–3 mm)	3.12 × 10^7^ ± 9.78 × 10^6^	2.13 × 10^6^ ± 1.07 × 10^6^	1.35 × 10^10^ ± 8.76 × 10^8^	3.79 × 10^5^ ± 2.47 × 10^4^
Layer 4, (3–4 mm)	7.05 × 10^7^ ± 2.81 × 10^7^	1.56 × 10^6^ ± 5.25 × 10^5^	5.35 × 10^9^ ± 4.51 × 10^8^	3.02 × 10^5^ ± 2.54 × 10^4^
Layer 5, (4–5 mm)	3.59 × 10^7^ ± 2.59 × 10^6^	9.62 × 10^5^ ± 1.95 × 10^5^	9.06 × 10^9^ ± 1.97 × 10^9^	3.71 × 10^5^ ± 8.05 × 10^4^
Layer 6, (5–6 mm)	7.90 × 10^6^ ± 3.55 × 10^6^	2.82 × 10^5^ ± 1.28 × 10^5^	3.24 × 10^9^ ± 4.50 × 10^8^	2.46 × 10^5^ ± 3.42 × 10^4^
Layer 7, (6–7 mm)	6.80 × 10^6^ ± 4.32 × 10^6^	6.41 × 10^5^ ± 4.81 × 10^5^	2.76 × 10^9^ ± 1.41 × 10^8^	2.37 × 10^5^ ± 1.21 × 10^4^

**Table 3:** Fungal taxonomic classification at phyla, class, order, family, and genera level. Average of the relative abundance (%) of the four layers. NA: no assigned at that level of classification.

Phylum	Class	Order	Family	Genus	Average%
*Ascomycota*	*Dothideomycetes*	*Capnodiales*	*Mycosphaerellaceae*	*Cercospora*	2.20
				*Zymoseptoria*	2.86
	*Eurotiomycetes*	*Eurotiales*	*Aspergillaceae*	*Aspergillus*	3.93
	*Leotiomycetes*	*Helotiales*	*Sclerotiniaceae*	*Botrytis*	4.39
	*Saccharomycetes*	*Saccharomycetales*	*Debaryomycetaceae*	*Candida*	4.62
				*Debaryomyces*	0.87
				*Scheffersomyces*	0.93
			*Dipodascaceae*	*Yarrowia*	2.16
			*Phaffomycetaceae*	*Komagataella*	0.84
			*Pichiaceae*	*Ogataea*	0.88
				*Pichia*	1.08
			*Saccharomycetaceae*	*Eremothecium*	2.11
				*Kazachstania*	1.50
				*Kluyveromyces*	1.69
				*Lachancea*	0.88
				*Nakaseomyces*	3.26
				*Saccharomyces*	1.77
				*Tetrapisispora*	2.74
				*Torulaspora*	0.99
				*Zygosaccharomyces*	0.64
			*Trichomonascaceae*	*Sugiyamaella*	1.14
	*Schizosaccharomycetes*	*Schizosaccharomycetales*	*Schizosaccharomycetaceae*	*Schizosaccharomyces*	0.88
	*Sordariomycetes*	*Sordariales*	*Chaetomiaceae*	*Thermothelomyces*	11.14
		*Hypocreales*	*Clavicipitaceae*	*Pochonia*	2.89
		*Glomerellales*	*Glomerellaceae*	*Colletotrichum*	7.00
			*Nectriaceae*	*Fusarium*	11.68
		*Magnaporthales*	*Pyriculariaceae*	*Pyricularia*	10.66
		*Sordariales*	*Sordariaceae*	*Neurospora*	4.72
*Basidiomycota*	*Tremellomycetes*	*Tremellales*	*Cryptococcaceae*	*Cryptococcus*	2.96
	*Malasseziomycetes*	*Malasseziales*	*Malasseziaceae*	*Malassezia*	0.92
	*Ustilaginomycetes*	*Ustilaginales*	*Ustilaginaceae*	*Sporisorium*	2.27
				*Ustilago*	2.94
*Microsporidia*	NA	NA	*Unikaryonidae*	*Encephalitozoon*	0.45

**Table 4:** Fungal genera which contributed the most to the global similarity of the community, according to SIMPER analysis. Average Abundance % (Av. Abund.), average similarity % (Av. Sim.), standard deviation (Sim./S.D.), contributed percentage % (Contrib.) and cumulative contribution % (Cum.).

	Av.Abund.	Av.Sim.	Sim./S.D.	Contrib.	Cum.
*Fusarium*	11.68	11.27	134.85	12.02	12.02
*Thermothelomyces*	11.14	10.58	19.09	11.29	23.31
*Pyricularia*	10.66	10.43	35.85	11.13	34.44
*Colletotrichum*	7	6.7	18.19	7.15	41.59
*Candida*	4.62	4.51	48.27	4.82	46.4
*Neurospora*	4.72	4.49	39.81	4.79	51.19
*Botrytis*	4.39	3.91	4.82	4.17	55.36
*Aspergillus*	3.94	3.72	12.63	3.97	59.33
*Nakaseomyces*	3.26	3.02	17.23	3.22	62.55
*Cryptococcus*	2.96	2.83	31.55	3.02	65.57
*Pochonia*	2.9	2.74	19.25	2.92	68.49
*Zymoseptoria*	2.86	2.6	22.96	2.78	71.27
*Tetrapisispora*	2.74	2.47	6.21	2.64	73.91
*Ustilago*	2.94	2.45	6.92	2.62	76.53
*Sporisorium*	2.27	2.26	274.52	2.41	78.94
*Yarrowia*	2.16	2.09	41.1	2.23	81.17
*Cercospora*	2.2	1.96	10.7	2.09	83.26
*Eremothecium*	2.11	1.93	6.64	2.06	85.32
*Saccharomyces*	1.77	1.64	20.02	1.75	87.07
*Kluyveromyces*	1.7	1.61	18.45	1.71	88.79
*Kazachstania*	1.5	1.4	12.92	1.5	90.28

## Supplementary Materials

The following are available online at https://www.mdpi.com/2309-608X/7/3/210/s1, Table S1: Read count and taxonomic classifications.
